# Phylogenetic networks empower biodiversity research

**DOI:** 10.1073/pnas.2410934122

**Published:** 2025-07-28

**Authors:** Sungsik Kong, Claudia Solís-Lemus, George P. Tiley

**Affiliations:** ^a^Wisconsin Institute for Discovery, University of Wisconsin, Madison, WI 53715; ^b^The Institute for Computational and Experimental Research in Mathematics, Brown University, Providence, RI 02906; ^c^Department of Plant Pathology, University of Wisconsin, Madison, WI 53706; ^d^Southern Genetics, LLC., Graham, NC 27253

**Keywords:** phylogenetic networks, gene flow, speciation, conservation biology, biodiversity

## Abstract

Reticulate evolution has long been recognized as a key mechanism that contributes to genetic and trait diversity. With the widespread availability of genomic data, investigating historical reticulate evolution across taxa has gained significant attention, driven by the rapid development of statistical methods for detecting nontreelike patterns. Phylogenetic networks provide a biologically intuitive approach to depicting evolutionary processes such as hybrid speciation and introgressive hybridization, which result in signatures of historical gene flow. Interpreting phylogenetic networks is especially critical for groups of conservation concern that lack reference genome resources and explicit hypotheses from prior investigation, such as those based on molecular data, morphology, or species distributions. Here, we highlight recent advances in computational methods for inferring networks from genome-scale data and offer guidelines for deriving biological insights from phylogenetic networks. Particular emphasis is placed on modeling hybridization and whole-genome duplication in the context of allopolyploidization. Practical recommendations for empirical studies and the limitations of commonly used methods are discussed throughout. We anticipate that phylogenetic networks will influence conservation biology and biodiversity research, emphasizing the need for careful consideration of reticulate evolution inferred from these networks in the near future. Networks will accelerate other pressing avenues of biodiversity research, especially investigations of orphan crops and climate change resilience in natural systems. The promise of phylogenetic networks connects with broader themes in the special feature Monitoring and restoring gene flow in the increasingly fragmented ecosystems of the Anthropocene by providing an emerging probabilistic framework for inferring historical connectivity between species and populations.

The evidence of reticulate, nontreelike processes across the Tree of Life is rapidly accumulating as the capacity to generate high-quality genomic data increases and methods for understanding the causes of gene tree variation, both in terms of gene tree incongruence and discordance (1), become more sophisticated (2–4). Phylogenetic networks, rather than strictly bifurcating phylogenetic trees, best represent these reticulate processes. Although phylogenetic networks are gaining traction in evolutionary biology and biodiversity research, challenges persist in “network thinking” including interpretation, method choice, model selection, and applicability to organismal groups whose life histories deviate from model assumptions.

The term “network” in the literature can refer to either explicit or implicit (or abstract) networks. Explicit networks provide a direct link between the biological processes driving variation in data and their interpretation (5). The underlying model in many phylogenetic network methods is an extension of the multispecies coalescent (MSC; 6, 7), known as the network multispecies coalescent (NMSC; see ref. 1 for introduction), that accounts for both incomplete lineage sorting (ILS) and reticulate evolution. Considering ILS and hybridization together is important, as both processes occur simultaneously and produce gene trees that do not match the species tree. The gene tree–species tree discordance due to ILS occurs because ancestral polymorphisms persist through multiple speciation events and are randomly fixed or lost in descendant lineages (*SI Appendix*, Fig. S1 *A* and *B*). Accounting for both ILS and hybridization can lead to significantly different expectations for gene tree variation compared to considering ILS alone (*SI Appendix*, Fig. S1 *C* and *D*). Understanding the expectations for gene tree variation under models that do and do not account for hybridization remains important as coalescent models play a prominent role in delimiting species or conservation units (e.g., ref. 8) as well as reconstructing the evolution of ecologically important traits (e.g., ref. 9).

Implicit networks summarize discordance based on distances among sequences or gene trees, regardless of the biological cause (10, 11). While implicit networks are a useful depiction of conflicting signals in data, explicit networks have more intuitive biological interpretations. A drawback of explicit networks though is that they were and still are computationally expensive, and this made implicit networks an attractive data exploration tool that could be complemented with hybridization tests, such as Patterson’s D-statistic (12) or other similar methods (13, 14). Hybridization tests capable of detecting non-ILS patterns for subsets of four lineages provided a practical approach for identifying reticulate events that could then be superimposed on a phylogenetic tree (e.g., ref. 15). While this two-step approach sparked exciting science and the continued development of hybrid detection methods (*SI Appendix*), multiple independent simulation studies agree that hybrid detection methods are sensitive to violations of their underlying assumptions (16–18) and perform poorly in cases of multiple reticulations (19, 20) or in the presence of ghost lineages (21, 22).

The advancement of scalable and robust methods for inferring explicit networks (23–28) has overcome some of the challenges of applying hybrid detection methods to a larger phylogenetic context while raising new ones. For example, Cui et al. (29) proposed a phylogenetic tree with gene flow events among *Xiphophorus* fishes (Poeciliidae) detected using hybrid detection methods. Note hybridization is often referred to as gene flow in the literature as the methods are agnostic about the consequences of gene flow. The depicted relationship, however, contradicted a reanalysis of the same data using phylogenetic networks (23). The inferred network detected fewer reticulation events than (29), and comparing the two results is difficult. It is possible that hybridization between common ancestors in the network explains the distribution of significant hybridization test results, or the network may have had false negatives due to constraints in the inference process. Similar observations (e.g., refs. 30–32) are being made across empirical research using phylogenetic networks, making it timely to provide more clarity on important aspects of network estimation such as the interpretation of inferred networks, model selection, and current limitations.

Phylogenetic networks will have a growing influence in biodiversity research as they have the potential to be highly inclusive in the study of reticulate evolution. They are effective with a few hundred loci and are improving in scalability. Such data are now readily obtained in a typical phylogenomic investigation that might include new species, museum specimens, and low-quality DNA. Networks will lack the fine-scale resolution of some demographic modeling approaches from population genomics (e.g., ref. 33) but can provide insights into the early stages of species discovery and be part of a process that advances appropriate groups to more in-depth population and functional genomics research.

## 1. Interpreting Phylogenetic Networks

Phylogenetic networks generalize phylogenetic trees by incorporating nontreelike evolutionary scenarios through reticulation (34, 35). The set of vertices in a network includes both speciation and reticulation events. In contrast to a tree node (*SI Appendix*), a reticulation vertex allows two incoming branches and one outgoing branch, representing a hybridization event that produces one hybrid descendant from two ancestors (*SI Appendix*, Fig. S2). Rooted phylogenetic networks (*SI Appendix*, Fig. S2*D*) explicitly depict directed evolutionary processes from the common ancestor represented by the root.

The reticulate evolutionary history is sometimes represented in a semidirected network (*SI Appendix*, Fig. S2*B*), obtained by suppressing the root of a directed network. Semidirected networks are thus unrooted, and the directionality of the edges (equivalent to branches in trees) is absent, except for the reticulation edges and its descendant branches (see ref. 36 for a formal definition). Consequently, semidirected networks are unable to determine the ancestor–descendant relationships between internal tree nodes, but the parental species–hybrid daughter relationship at a reticulation vertex remains valid. Although semidirected networks can be tricky to interpret biologically, particularly where directionality is ambiguous, they show favorable identifiability results under several models (37–39). In other words, the parameters in these networks can be accurately inferred given the infinite amount of data, in theory. Semidirected networks are under active research from a variety of directions including the fundamental theory that can facilitate network searches (36) and combinatorial methods for network reconstruction (40, 41). Unrooted, undirected networks (i.e., implicit networks) are unsuitable for evolutionary investigations due to their phenetic assumptions, lack of directionality in the topology, and the significantly different topologies frequently produced compared to conventional Bayesian methods (10, 11, 42). Instead, implicit networks are useful for understanding conflicting biological signals in the data, providing general insight into underlying processes in a very short time, and visualizing those conflicts.

At a reticulation vertex, the genetic material is horizontally inherited from the parental species to the hybrid daughter. The proportion of genetic material that traces back from the hybrid daughter to a parent is denoted by the inheritance probability, commonly expressed as *γ* (43). This is distinguished from the migration rate, which denotes the rate that individuals from one population move and interbreed with another population. The inheritance probability is assigned to one of the two incoming edges at a reticulation vertex (*SI Appendix*, Fig. S2 *B* and *D*). Note that the value of *γ* lies between zero and one, meaning the inheritance probability of the other incoming edge toward the same reticulation vertex is 1−γ. The length of a reticulation edge can be zero when the sampling of parental species is complete (i.e., parental species are included in the data). Nonzero edge lengths can occur in cases of parental species extinction, incomplete taxon sampling, prolonged continuous introgression, or the presence of ghost lineages that played a role in the reticulation history (*SI Appendix*, Fig. S2*D*).

When γ≈ 0.5 at a reticulation vertex, the two parental species contribute fairly equally to the hybrid offspring (i.e., symmetrical hybridization). This is particularly expected in a diploid $f_{1}$ hybrid. The hybrid lineage may maintain *γ* close to 0.5 in subsequent generations if it successfully evolves into a distinct hybrid species through sustained reproduction among hybrids (i.e., hybrid speciation; 44). However, γ≈ 0.5 does not necessarily indicate hybrid speciation with no backcrosses to the parental species as the underlying process. An alternative model, such as bidirectional backcrossing of the hybrid with both parental species at equal rates, can also result in *γ* close to 0.5.

Hybrids may backcross with one of the parents in a more unidirectional manner (i.e., introgressive hybridization). Through repeated backcrossing, the hybrids acquire more genetic material from the parent with which they backcross (i.e., asymmetrical hybridization). While signals of symmetrical hybridization are easier for computational methods to detect, much of the literature on hybridization has favored asymmetrical hybridization as being more common in nature (e.g., *Carpinus* sect. *Distegocarpus*; 45). Sometimes γ≈ 0.5 or very close to 0 or 1 are interpreted as recent or ancient hybridization events, respectively (46), particularly when hybrid detection methods that do not consider the timing of the event are used to estimate *γ*. An arbitrary threshold is used to draw a line between recent and ancient hybridization [e.g., 0.3<γ<0.7 for recent hybridization, and ancient otherwise (47)]. However, distinguishing between hybrid speciation and introgressive hybridization using network methods alone or between ancient and recent hybridization events using the value of *γ* is challenging and may involve subjectivity. Additional genomic information from high-quality assemblies may be helpful, but ideally other lines of biological evidence such as the source of reproductive isolation would be available.

**Fig. 1.**
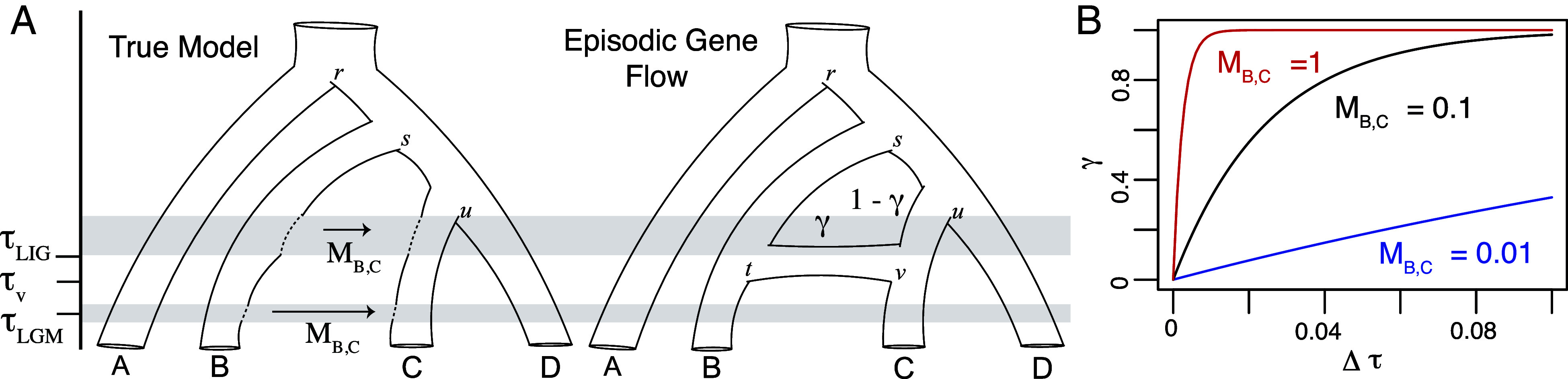
Networks with episodic gene flow approximate more complex processes. (*A*) A model of continuous gene flow looking backward in time considers that genes trace from lineage C into lineage B at a rate of $M_{B,C}$ immigrants per generation over the intervals in gray that occur before $\tau_{\mathrm{LIG}}$ (The Last Interglacial Period) and around $\tau_{\mathrm{LGM}}$ (The Last Glacial Maximum). Such observations are not infrequent in nature as paleoclimate cycles can change species distributions and create ephemeral contact zones. Species networks that use either gene trees or sequences as data will assume that genes trace back from lineage C into B at time $\tau_{v}=\tau_{t}$ with probability *γ*. Here, $\tau_{v}$ is the time of hybridization under a phylogenetic network or episodic model. The assumption that $\tau_{v}=\tau_{t}$ can be relaxed, but in this case $\tau_{v}$ will fall between $\tau_{\mathrm{LIG}}$ and $\tau_{\mathrm{LGM}}$. Were there a single interval with a constant effective migration rate $M_{B,C}$, $\tau_{v}$ would be the midpoint of that interval. (*B*) The expectation for *γ* under the isolation-with-migration model is determined by the effective migration rate over the interval that migration occurs, Δτ. Plotting *γ* as a function of Δτ for different migration rates used an expected pairwise genetic distance (*θ*) between two individuals sampled from the receiving lineage of 0.01. The relationships between the inheritance probability (*γ*), migration rate (*M*), and time (*τ*) shows that interpreting the age of hybridization and rate of gene flow from a network can be difficult when the true model is a more complex demographic process, such as the scenario in panel *A*.

A caveat of phylogenetic networks is that all reticulation events are episodic. An episodic model may be an oversimplification of the process as multiple individuals over multiple generations are likely necessary in establishing a viable population (Fig. 1*A*) and gene flow might happen in multiple pulses, which can coincide with paleoclimate cycles. Such phenomena are well documented in mountain ranges whether the Andes (48) or the Alps (49). However, the assumption provides computational convenience for network searches compared to the isolation-with-migration (IM) model (50, 51) that integrates over gene genealogies to estimate continuous migration rates. Some important intuition about the inheritance probability *γ* can be developed from IM models though. If continuous gene flow happens at a rate of *M* effective migrants per generation over a time interval Δτ, then $\gamma =exp\left\{\frac{4M\Delta \tau }{\theta }\right\}$, where *θ* is the effective population size (52). This shows that it is possible to arrive at values of *γ* near 0.5 from continuous processes that do not invoke hybrid speciation (Fig. 1*B*) and that caution is needed in regard to interpreting *γ* as evidence for hybrid speciation versus introgressive hybridization.

## 2. Estimating Phylogenetic Networks

In this section, we discuss computational approaches to estimate phylogenetic networks from empirical data. Inferring networks requires greater computational effort than estimating trees due to the enormous multidimensional network space and a greater number of parameters in the model. Methods that infer a network directly from sequence data (i.e., full data methods) are powerful as they remove the effects of gene tree error on network estimation and make it possible to identify models not detectable by other methods such as gene flow between sister species or ring species. However, these methods are computationally heavy. To ameliorate the computational burden, some methods take a two-step strategy that summarize the sequence data into another format (e.g., gene trees) prior to estimating the network (i.e., summary methods). In addition to practical considerations over how a model is implemented, there is now a suite of options (*SI Appendix*, Table S1) that address different biological processes under different optimality criteria.

### 2.1. Hybridization.

Under NMSC, two biological sources of gene tree incongruence are ILS and hybridization (*SI Appendix*, Fig. S1). These two processes co-occur in nature and should be considered jointly by phylogenetic networks. Some methods infer networks assuming the absence of ILS [e.g., NETRAX (53)] and this can be appropriate when the interval between speciation events is large, but most organismal studies likely need to consider both. For example, when wanting to understand the processes of speciation and evolutionary history of a rapid radiation, short speciation intervals (that will increase the amount of ILS in data) and introgression are often a packaged feature of the group (54), as has become well-appreciated from studies of African cichlids (e.g., ref. 55).

PHYLONET (24) is arguably the most popular software for analyzing, reconstructing, and evaluating phylogenetic networks (see ref. 25 for a detailed summary of the main functions). The PHYLONET function InferNetwork_MP (56) infers maximum parsimony (MP) networks from a set of gene trees. While InferNetwork_MP can handle a large number of tips very efficiently, the estimation accuracy is lower than that of likelihood-based methods (57) and lacks statistical consistency in some biological scenarios such as long branch attraction. PHYLONET also implements InferNetwork_ML (58), which infers a maximum likelihood (ML) network from a set of estimated gene trees with or without branch lengths. Estimating ML networks is computationally demanding and may take more than a week for a dataset with ten tips, for instance (23, 57). One way to ameliorate the computational burden is to directly use the branch lengths in the input gene trees during estimation; although, inaccurately estimated branch lengths can add unwanted error. Refer to *SI Appendix* for details on the Bayesian approaches implemented in PHYLONET, as well as other methods available in BEAST2 and BPP.

Methods that use composite likelihood (CL; 59) have improved the scalability of network inference. InferNetwork_MPL implemented in PHYLONET uses the product of likelihoods across rooted triplets (i.e, three-taxon trees) from a collection of gene trees, and widely applied to empirical systems (e.g., refs. 60–62). However, InferNetwork_MPL is not guaranteed to recover the true network since the same set of triplets could be expected by multiple networks. A CL approach for biallelic marker data is also available in PHYLONET, MLE_BiMarkers (63). SNaQ (Species Network applying Quartets) (23), originally implemented in the JULIA package PHYLONETWORKS (64) but now its own package, is similarly used across a wide range of taxa (e.g., refs. 65–67). The CL of a network in SNaQ is approximated by calculating the product of the likelihoods of unrooted quarnets (i.e., undirected networks with four taxa; *SI Appendix*, Fig. S2 *A* and *B*) within the network, using quartet concordance factors [CFs; the proportion of the genome supporting a given clade (68)]. The CF distribution is typically computed directly from a set of gene trees (69) but can also be derived from other sources such as a Bayesian posterior samples of gene trees (70) or biallelic marker data (71). A separate JULIA package PHYNEST (Phylogenetic Network Estimation using SiTe patterns; 26) estimates networks directly from sequence alignments with the function phyne!. PHYNEST focuses on rooted quartets in the displayed trees of a network for its CL computation and infers a rooted species network using an outgroup. PHYNEST implements simulated annealing algorithms [inspired by Salter and Pearl (72)] for network space traversal, which enhances the chance of finding the global optimum compared to hill climbing. We briefly described a portion of the existing methods, but see *SI Appendix*, Table S1 for other approaches not discussed in detail.

A major caveat is that the field is under active development and while the potential for networks in evolutionary biology and biodiversity research is appreciated, failures of network methods in empirical settings are notable. For example, Thawornwattana et al. (73) reported their attempts to apply MCMC_SEQ and InferNetworks_ML in PHYLONET and SNaQ in PHYLONETWORKS to a well-characterized genomic dataset of *Heliconius* butterflies (74) were unsuccessful as the networks estimated in different independent runs were inconsistent and contained biologically spurious reticulations. An analysis of 14 fur seal and sea lion genomes (Otariidae) (75) attempted to use BEAST2 (SPECIESNETWORK) and PHYLONET (MCMC_GT and MCMC_SEQ) but were unsuccessful as results were inconsistent across methods and runs had apparent convergence difficulties. Similar inconsistencies were reported for both full likelihood and CL analyses of five ruminant families with 10,000 loci (30). The families were not too old, having diversified through the Miocene, but the taxon sampling would have made identifying a network with more than one or two hybrid edges impossible (76). The same statistical identifiability issues were probably encountered in a study of seven Old World Monkey species within Papionini (31). The authors found that PHYLONETInferNetwork_MPL and SNaQ returned biologically ambiguous networks, but the multiple anticipated hybridization events were likely beyond limitations of the sampling and models (76).

Most network methods are still restricted to level-1 networks, which means that the reticulations are isolated from each other (5, 34). For methods that do not assume networks are level-1, recovering the correct network with nested reticulations can be difficult as demonstrated by an investigation of diploid wheat relatives (77). The inference of networks that relax the level-1 assumption and their statistical properties are under active development and have recently been applied to a species-rich clade of *Desmognathus* salamanders (Plethodontidae) from the southern Appalachians (78). However sampling taxa to restrict anticipated networks to level-1 should be helpful as they are known to be identifiable with both full likelihood and CL (39, 79).

### 2.2. Allopolyploidy.

Most, if not all, methods discussed so far assume species are diploid. It is not uncommon across plants and observed in some animal groups such as amphibians (e.g., ref. 80) and fish (e.g., ref. 81) to have more than two copies of the genome, polyploidy. The science of polyploidy has long captivated botanists and it is not entirely independent of hybridization, as hybridization is frequently a pathway to polyploidization. Polyploids that arise through hybridization between distinct species, sometimes resulting in distinct subgenomes (82), are allopolyploids and they represent about half of known polyploid species diversity (83). Allopolyploids with two genetically distinct parental lineages can be appropriately represented by networks (84, 85). However, polyploid networks have a slightly different interpretation from the diploid case as the polyploid hybrid daughter inherits the entire genetic information from both parents and contains multiple full sequences. Note γ=1 at every reticulation edge for polyploid network as entire genome from both parents are inherited to the hybrid. Thus, inheritance probabilities might represent biased fractionation (86) rather than backcrossing. Nevertheless, phylogenetic networks have been applied to several empirical cases of allopolyploidy such as ref. 87 that applied InferNetwork_ML of PHYLONET to a dataset containing both diploid and polyploid (ranging from tetraploid to decaploid) species in *Fragaria*. The authors showed that while InferNetwork_ML is not originally developed to model allopolyploid speciation, it can accommodate assigning multiple haplotype sequences to the allopolyploid species, and this approach should generally work for recent allopolyploids that avoid confounding deep paralogy from ancient whole-genome duplication with the hybridization signal (13). Allopolyploids can raise many challenges to the NMSC as they may be part of a larger complex where parental diploids contribute to multiple polyploid lineages, and the application of phylogenetic networks to polyploid complexes in the absence of multiple lines of evidence may be misleading (88, 89). The NMSC also ignores an important piece of information, the ploidy of the species under investigation. Multiple methods are now available, with more in development, that use such information to improve network searches and bring more meaningful biological insights.

The first explicit allopolyploid model was ALLOPPNET (90–92), a fully parameterized stochastic model implemented in ^∗^BEAST (93). Using the multilocus DNA sequence data, ALLOPPNET uses MCMC to sample the posterior distribution of phylogenetic network topology, ages of speciation and allopolyploidization, and population sizes. ALLOPPNET’s model allows biologists to explore a wide range of scenarios by constraining the divergence times to force simultaneous transfer of homeologs from the two putative diploid parents toward a tetraploid daughter. ALLOPPNET was successfully applied in some empirical systems, such as revealing the formation of an allotetraploid fern in Cystopteridaceae where the parents shared a common ancestor nearly 60 mya (94), or resolving two tetraploid *Medicago* (Fabaceae) species as having a shared allopolyploid origin (95). The model has limitations though, as it only accounts for tetraploids that arise from two diploid parents and requires a priori assignment of haplotype sequences to subgenomes. The subgenome assignment problem is increasingly trivial with long read sequencing and subgenome identification pipelines (96), but such sequencing effort is not always possible and higher ploidy levels can be encountered in many taxonomic groups.

Instead, fast parsimony methods that leverage ploidy information to search for only biologically plausible networks are promising. InferNetwork_MP_Allopp (97) in PHYLONET uses a collection of gene trees and an extension the Minimizing Deep Coalescences criterion (56) that generalizes the method of GRAMPA (98) by allowing ILS. This approach builds off of methods that would first estimate a multilabeled species tree that was used to infer the species network (*SI Appendix*). Polyploid biology then comes under consideration. InferNetwork_MP_Allopp allows users to assign haplotypes to subgenomes a priori, similar to ref. 90, or let heuristics map haplotype sequences to their corresponding subgenomes. A taxa map that contains information on known hybrid species is also used to constrain the network space such that the leaves below the reticulation vertex only contain the known hybrid species. The taxa map information should then prevent the proposal of biologically unrealistic networks where, for example, a diploid hybrid has polyploid parents. Using data from hexaploid and tetraploid wheat (99), InferNetwork_MP_Allopp was shown to obtain the expected relationships. Reassuring results were also obtained from a case study in Asteraceae (100), but only when the allopolyploids were identified in the taxa map. Analyses of a genus in Brassicaceae with low sequence divergence (101) and ancient allopolyploidy events in Malvaceae (102) failed to recover anticipated relationships. These studies reinforce important aspects of successful network analyses in general, for example, the parental species need to be distinct in the gene trees but not so old that error or other sources of gene tree variation confound the signal. The method of Yan et al. (97) represents important steps that incorporate aspects of polyploid biology into network searches that can be considered for other approaches as well.

## 3. Guidelines for Applying Network Approaches

In this section, we provide general recommendations for applying network approaches to empirical data. We assume that taxa in the dataset are suspected to have experienced reticulate histories supported by biological information and potentially other analyses.

### 3.1. Data.

A variety of data types can be used as input for network analysis (*SI Appendix*, Table S1). The formatting of multiple sequence alignments or biallelic data depends on the method. For example, methods in PHYLONET use the NEXUS file format, PHYNEST uses the PHYLIP format, and SpeciesNetwork requires the NEXUS file to be converted into a BEAST XML input file using BEAUTI (103). The input for summary methods is typically a list of gene trees estimated from conventional tree methods, which may be unrooted or require rooting. The accuracy of input gene trees affected by sequence quality, rooting errors, misidentification of the substitution model, and orthology errors directly influences the performance of summary methods. Full data methods should be robust to low-information sequences provided enough loci are sampled, but the sensitivity to, for example, a small proportion of orthology errors has not been explored.

Increasing the number of loci in the data may improve the accuracy of estimates; however, this benefit depends on the evolutionary history and the scope of analysis. A single recent hybridization event among a few taxa can often be reconstructed from a few to dozens [e.g., four loci were sufficient for the Cystopteridaceae case (94)], while hundreds to thousands of loci may be necessary to infer larger and more complex networks. Computational time also increases with the number of taxa for all methods, although the degree varies based on the optimality criteria used. For CL methods, the running time remains relatively constant with additional loci, but it grows exponentially for full likelihood methods, becoming prohibitive even with datasets of 300 loci for 10 taxa (23). In general, <10 taxa for full likelihood methods and <25 for CL methods is considered reasonable (*SI Appendix*, Fig. S3; 57). If only estimating parameters on a fixed network is the goal (as in the MSCi model in BPP, for example), then larger analyses with full likelihood might work. A reanalysis of *Jaltomata* transcriptomes in ref. 32, that used 1000 loci and 14 species, required 3 to 4 d per MCMC run with eight cores per run, for example.

### 3.2. Analysis.

Regardless of data type, there will be a heuristic search for the best network. Most methods conduct five (e.g., InferNetwork_ML) to ten (e.g., InferNetwork_MPL, SNAQ, PHYNEST) independent runs per analysis by default. However, in practice, more than ten runs (e.g., refs. 104 and 105), up to one hundred in extreme cases (106), are conducted to find the best network, particularly when there are many taxa with multiple anticipated reticulation events. The network estimated by a single run could be a local optimum. The appropriate number of runs depends on the number of tips and computational capacity as multiple cores enable the parallelization of many independent runs. All searches also depend on a starting tree (*SI Appendix*), and multiple runs with different starting trees might be helpful.

The number of reticulations expected in the final network (*h*) often needs to be specified prior to the analysis. For methods that infer level-1 networks, the upper limit of *h* in a topology with *n* tips is computed as $\left\lfloor \frac{n-1}{2}\right\rfloor$ (107, 108). In practice, unless *h* is known or supported by biological or external evidence, a series of network analyses are conducted for a range of *h* values. Post hoc analyses are then used to pick the best value of *h*.

### 3.3. Postanalysis.

**Fig. 2.**
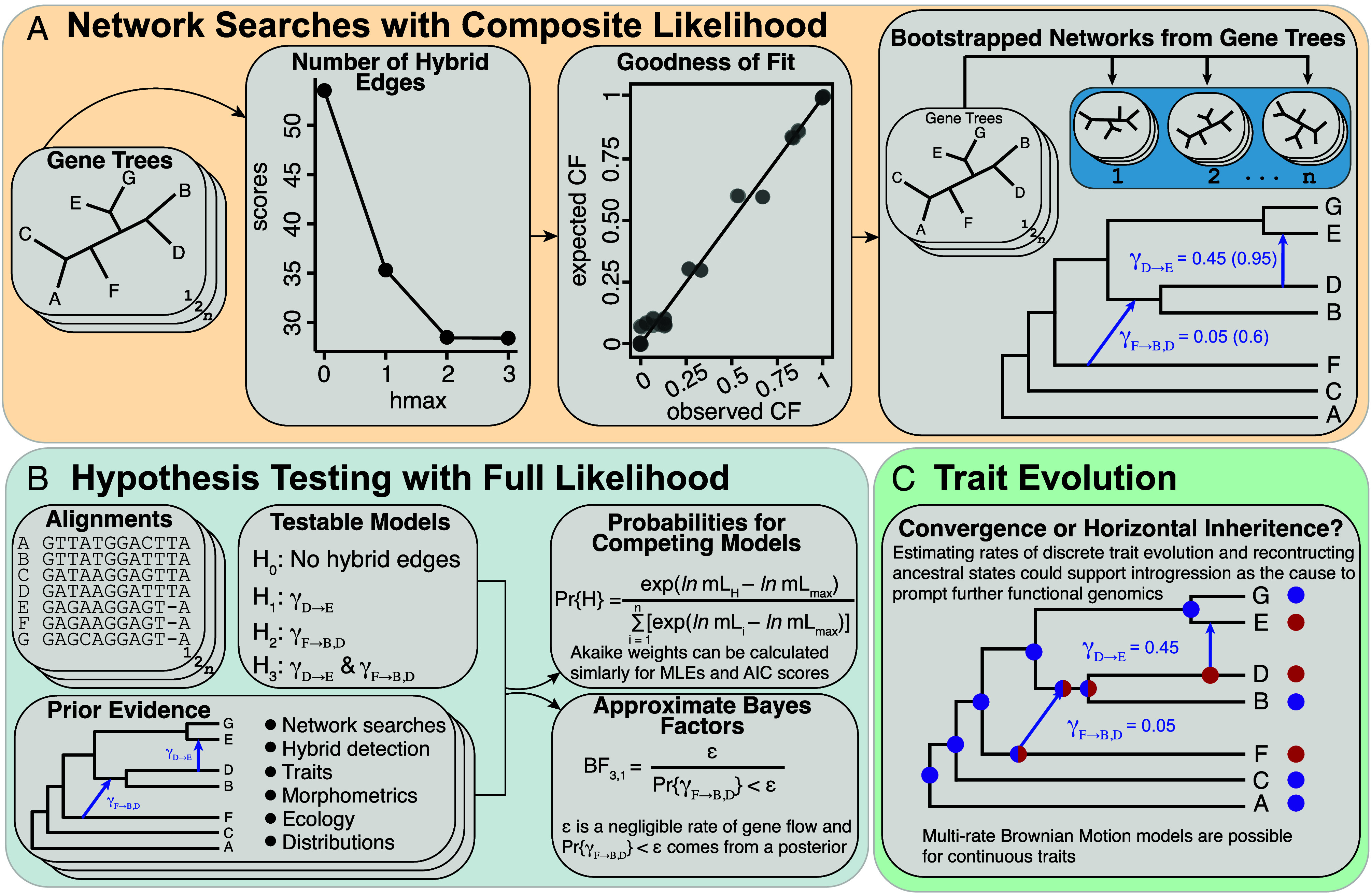
(*A*) We focus on the network analysis that starts with gene trees as input data using composite likelihood. Because composite likelihoods are not comparable with typical full likelihood techniques such as the Akaike information criterion (AIC), heuristics are used to determine the inflection point where increasing the number of allowed reticulation edges adds little value. Tools are available for evaluating the quality of an estimated network. For example, one could check the goodness of fit by comparing the observed versus expected concordance factor distribution. A random cloud of points would show that the network explains the data poorly and other models or sources of gene tree variation should be considered. It is possible to obtain bootstrap support values for reticulation and tree edges in a network; this entails resampling gene trees and estimating networks for each sample to obtain the edge supports. Bootstrap support values for networks are not well-studied like binary trees, so it is difficult to suggest hard cutoffs. (*B*) Further hypothesis testing is possible when the number of tips are not too large. Here, network searches are usually not done under full likelihood but the sequence data can be used directly to support one over other plausible scenarios. A network may come from composite likelihood analyses, hybrid detection methods, or other sources of information such as morphology. Model probabilities can be obtained with either AIC scores in the case of maximum likelihood or using the log-marginal likelihoods from Bayesian Markov chain Monte Carlo sampling. Obtaining the log-marginal likelihoods from Bayesian analyses is computationally expensive and alternative techniques are possible for nested hypotheses that use a single posterior sample directly. (*C*) Once there is some confidence in a network for a group, models of trait evolution can be applied to estimate the probability that a trait distribution was due to introgression. All of these analyses happen at the species level for a sample of phylogenomic loci, which provides opportunity for downstream functional genomic investigations for candidate traits of interest.

Given a set of networks with different *h*, all estimated from the same data, how do we select the best one? For full likelihood methods, information criteria (109, 110) or K-fold cross validation can be used (58). However, such approaches might not be appropriate for CL methods. We anticipate that the CL score will always improve with more reticulations and determining what is a meaningful improvement requires some subjectivity. One can visualize the CL score as *h* increases and select *h* where the drop in composite likelihood value flattens out (Fig. 2*A*). Such slope heuristics have been used for model selection in network inference (23). Goodness of fit tests are also available for phylogenetic networks (111) through the JULIA package QUARTETNETWORKGOODNESSFIT, which constructs a z-score to provide an investigator with a p-value based on the observed versus expected CFs from a collection of gene trees. If the differences between two CF distributions is large, there are likely other factors, both technical and biological, that require consideration before interpreting the network (Fig. 2).

Bootstrapping for networks is possible, but summarizing a set of bootstrap networks can be tricky. Since edges in networks do not uniquely define splits of taxa, frequencies of splits among the bootstrap replicates cannot be used to quantify support for a given edge in a network. In PHYLONETWORKS, support is quantified for individual hybridization events through the presence of three relevant clades: the hybrid clade (descendants of hybridization event), major sister clade (connected to the hybrid clade through the major edge with γ>0.5), and minor sister clade (connected to the hybrid clade through the minor edge with γ<0.5).

Once the network or set of candidate networks has been selected, they can be used for comparative methods (Fig. 2*C*), such as evaluating the evidence for transgressive evolution of traits (35). For example, a discrete trait model used to show introgression as opposed to convergence was better explanation of flower color among Malagasy baobabs (*Adansonia*: Malvaceae) (65). A continuous trait model was applied to geometric morphometric data from Patagonian *Bariupus* ground beetles (Carabidae) to show transgressive evolution of a potentially adaptive trait associated with burrowing ability (112).

### 3.4. Hypothesis Testing.

Most of our discussion has emphasized the search for the optimal network under various criteria. Sometimes, a specific group benefits from a great deal of organismal expertise and prior analyses, such that only a small collection of candidate networks (i.e., hypotheses) are plausible (Fig. 2*B*). In such scenarios, we can compare models with ML (58) using information criteria such as Akaike information criterion (88) or obtain model probabilities (113) from Bayesian methods (114). The Bayesian methods require estimating marginal likelihoods, which incur a heavy computational burden requiring multiple MCMC chains for each hypothesis (32, 89). The performance of different integration techniques for marginal likelihood estimation with networks has not been well explored like binary trees (115). An alternative approximation of Bayes factors for nested hypotheses is possible with the Savage–Dickey density ratio (32, 116). This test appears promising as it does not require separate analyses and many MCMC runs for each model and can reject negligible signals of introgression or accept a more complex case not easily unveiled with hybrid detection methods or level-1 networks.

## 4. The Potential for Networks to Accelerate Biodiversity Research

Much of biodiversity science starts from the fundamental phylogenetic systematics research. For example, collections must be made and species delimited and described before that species and records are available in the Global Biodiversity Information Facility for large-scale distribution modeling applications. The increasing availability of phylogenomic data creates opportunity for phylogenetic networks to be as common as the trees, when appropriate. There are three practical challenges that impede, for example, a study of a genus or multiple closely related genera with a few hundred species: 1) the computational burden, 2) known statistical identifiability concerns, and 3) an ever-present fear of false positives. There is no single analysis that will be satisfying for tackling the group at once, but a holistic approach that uses networks in conjunction with existing phylogenomics best practices should help progress investigations of reticulate evolution within the group (Fig. 2).

### 4.1. Applications to Conservation Science.

Mitigating extinction risk is a serious concern for many biodiversity scientists. Across vertebrates, some of the most recently described taxa are those most at risk (117). The number of extinctions and risk categories across plants has also increased from local habitat loss and global trends in anthropogenic climate change, and sometimes in unintuitive ways such as cactus species (Cactaceae) that are susceptible to increased temperatures despite their drought resistance (118). Because the proportion of threatened species is distributed unevenly across taxonomic groups and geographic regions (119), it can be difficult to craft conservation policy that prioritizes both regions of high species endemism such as *biodiversity hotspots* (120) and phylogenetic diversity (121). A better understanding of reticulate evolution may help set these priorities. For example, signals of historical gene flow coupled with a suite of species delimitation techniques were used to synonymize multiple species of mouse lemurs (*Microcebus*: Cheirogaleidae) (8), and the taxonomic recommendations would remove several endangered species and potentially have downstream effects regarding which forests should be prioritized for protection. Simply recognizing some amount of historical gene flow does not obviate the need for nuanced policy though. In the case of giraffes (*Giraffa*: Giraffidae), a debate is whether one species should be recognized as at least four species with multiple conservation units to safeguard genetic diversity (122). Thus, understanding the history of reticulate evolution within a group is not opposed to conservation goals but may provide more context for understanding species distributions and patterns of genetic variation. The cases of mouse lemurs and giraffes are driven largely by population genomic investigations, but species networks should help identify interesting cases within larger phylogenomic investigations that can be advanced to population genomics and demographic modeling for communication with conservation biologists and managers.

### 4.2. Finding Traits Conferring Climate Change Resilience.

Allopolyploid species complexes in plants have been a source of genetic and trait diversity subjected to selective breeding in agriculture. Allopolyploids can push quantitative traits beyond the distribution observed in either parental lineage, such as oil production in rapeseed (123), fiber length in cotton (124), or combinations of advantageous qualitative traits like those in free-threshing wheat (99). Wheat represents a complex of allotetra- and allohexaploids where the mechanisms (125) are contentious, but large-scale genomic data combined with phylogenetic networks have helped elucidate the evolutionary history of bread wheat and the subgenome origins of threshing traits (9).

The aforementioned allopolyploid plants were well-characterized systems by the time phylogenetic networks arrived, but their true power will be resolving the evolutionary history of emerging or orphan crops. These are species that have undergone some degree of domestication but lack international utilization sometimes due to drawbacks with respect to yield or ease of processing [reviewed by Dawson et al. (126)]. However, orphan crops can have traits of acute interest to climate change resilience. For example, Fonio millet (*Digitaria exilis* Stapf) is an allotetraploid cereal crop found in Western Sub-Saharan Africa that is adapted to drought stress and soils with a low organic nutrient content (127, 128). However, there remains great uncertainty in the parental lineages and genetic basis of desirable traits. Networks offer a way forward for resolving the reticulate history of orphan crops with respect to their wild relatives while lacking the genomic resources of staple crops such as wheat. Modern agriculture currently exploits about 200 domesticated species of about 2,500 that have undergone some form of domestication (129), and changing this will require accelerating the pipeline from discovery to functional genomics and breeding programs.

Outside of an agricultural context, introgressive hybridization has been implicated in the rapid acquisition of traits advantageous under low precipitation conditions such as C_4_ photosynthesis (130, 131). Given the numerous examples of reticulate evolution across the plant Tree of Life recently documented [reviewed by Stull et al. (4)], the resources to ensure ecosystem services are resilient to climate change likely exist in natural systems. For example, the impending availability of chromosome-level genomes for weeds across multiple plant groups provides opportunity to understand contributions of hybridization and polyploidy to the genetic basis of tolerance to a range of environmental factors (132), including recently introduced anthropogenic ones such as herbicides (45). High-quality phylogenetic networks coupled with models of discrete and continuous trait evolution (35, 64) should be able to efficiently guide functional genomics experiments aiming to translate the genetic basis of resilience traits into material and biodiversity gains.

### 4.3. Challenges to Aligning Network Methods with Biodiversity Goals.

Substantial progress has been made on the tools used to estimate phylogenetic networks and their scalability. Matters of scale will continue to be overcome whether through divide-and-conquer techniques (133), improved optimization (134), or graphics processing unit acceleration similar to other phylogenetic likelihood calculations (135). Statistical properties of networks are still under investigation (23, 39, 76, 79, 89), so specific guidance on any one model or best practices will be dynamic, but biodiversity and conservation scientists may need to start planning for how networks will affect their practices and decision making. For example, some biodiversity analyses will prioritize phylogenetic diversity in risk assessments (136), but network-based diversity metrics (137, 138) may need to be considered, especially for groups where parental lineages of hybrids can be deeply diverged as observed in ferns (94). Conservation practitioners may be faced with scenarios where a rare species with a limited distribution is revealed to have a history of gene flow with a widely distributed species, is it still deserving of protection and training others to identify it and report occurrences? Genetic diversity is generally appreciated but not a factor in determining International Union for Conservation of Nature Red List status, so perhaps good policy would ignore networks and leave it to systematists to develop a meaningful and accepted taxonomy for their respective groups? There can be many positive outputs from networks though, such as rapidly advancing our ability to identify areas of larger phylogenomic studies that should be subject to more nuanced population genomics or functional genomic investigations. This creates opportunities for biodiversity scientists and method developers to work together on advancing research that prioritizes resilience traits or other features conferring resilience to climate change and generally detrimental anthropogenic effects.

## Supplementary Material

Appendix 01 (PDF)

## Data Availability

There are no data underlying this work.
