# Supplementary material for: Phylogenetic networks empower biodiversity research
Source: Proc Natl Acad Sci U S A. 2025 Jul 28;122(31):e2410934122. doi: 10.1073/pnas.2410934122 (PMC12337313; doi:10.1073/pnas.2410934122)
Supplement: Supplementary file 1 — Appendix 01 (PDF) [file pnas.2410934122.sapp.pdf]

Supporting Information for

Phylogenetic Networks Empower Biodiversity Research

Sungsik Kong, Claudia Solís-Lemus and George P. Tiley

George P. Tiley.
E-mail: george.tiley@gmail.com

This PDF file includes:

Supporting text
Figs. S1 to S3
Table S1
SI References

Supporting Information Text

Hybrid detection methods. Hybrid detection methods detect signals of historical gene flow between species or populations without inferring their relationships. A Python package HyDe (Hybrid Detection; (1)), the Patterson's D-statistic (the ABBA-BABA test; (2)), D_3 (3), and D_p (4) use site pattern frequencies or genetic pairwise differences to test the null hypothesis of tree-like evolution within a predefined subset of three or four taxa. The D-statistic (and related) tests are intended as tests for introgression between two species irrespective of the direction of gene flow, while HyDe tests for hybrid speciation of a third taxon originating from two parental taxa. MSCquartets (5) is an R package (6) that takes estimated gene trees as input, from which it computes the observed quartet counts concordance factors (qcCFs; (7)) which are compared to the expected concordance factor (CF) values obtained under the MSC. Deviations from the MSC are interpreted as evidence of hybridization signal. MSCquartets does not require a specific tree topology to test the CF expectations against, instead utilizing the information on all three qcCFs per four-taxon subset to compute the test statistic. PhyloDiamond (8) is a recent hybrid detection method that specializes in detecting 4-node hybridization cycles from algebraic invariants on qcCFs. It is not a hypothesis test as it uses algebraic invariants to identify places in the topology where there is signal for a four-node hybridization cycle (e.g., Supplementary Figs.S2b and S2d; the reticulation composed of four nodes). None of these tests detect gene flow between sister taxa as that will not disrupt the expected frequencies of site patterns or distances expected under ILS alone S1.

Terminology for phylogenetic trees and networks. Phylogenetic trees are a special case of explicit networks in which the topology contains no reticulation (Supplementary Fig. S2). A (binary) tree represents the genealogical relationships among a set of taxa as a strictly bifurcating branching structure, with the assumption that all speciation events represented by bifurcations produce exactly two descendants from a common ancestor. Trees assume vertical evolution, where genetic material is inherited directly from ancestors to descendants. A tree consists of a set of nodes (which include internal and terminal nodes, or leaves) and a set of branches, and can be either unrooted (Supplementary Fig. S2a) or rooted (Supplementary Fig. S2c). If rooted, it has a unique node called the root, which transforms the unrooted branching diagram into an evolutionary hypothesis by providing directionality from the root toward the leaves. The leaves represent sampled extant species (or fossil species in the case of a fully extinct clade), while the internal nodes and root are inferred from data and represent the ancestors of all taxa descended from that node.

The set of branches that connects two nodes in a tree represents the pathway of genetic transmission between the nodes. Suppose there is a branch between nodes u and v that is directed from u to v (or tail to head; Supplementary Fig. S2c). Here, u is either the root or an internal node, and v is either an internal node or a leaf. Biologically, u is interpreted as the ancestor of v, and v is the descendant of u. Each internal node has a node age each of which represents the time of the corresponding speciation event, and the branch length is the absolute difference between the ages assigned to u and v. As mentioned, the units of branch length depend on the model and the optimality criteria used to fit the data to the tree. Commonly used units include coalescent units (Supplementary Fig. S1) or the number of substitutions per site. When root-to-tip lengths are identical for every leaf, the tree is said to be ultrametric.

Bayesian methods for phylogenetic network estimation. Functions `MCMC_GT` (9), `MCMC_SEQ` (10), and `MCMC_BiMarkers` (11) are the Bayesian approaches implemented in PhyloNet. `MCMC_GT` uses a set of gene trees as input, where each locus can have one or more topologies. Two priors to fully specify the model are: (1) on the phylogenetic network consist of topology, branch lengths, and number of reticulation nodes and (2) on the inheritance probabilities for each reticulation. While selecting an unreasonable prior will be detrimental to the inference, selecting a suitable prior for a specific dataset is challenging (see below). In all three Bayesian approaches, posterior probabilities are calculated from different dimensions in model space using a reversible-jump Markov chain Monte Carlo algorithm (12).The computational bottleneck of Bayesian inference is more severe than the ML inference and can take weeks or more to complete the analysis even for a relatively small dataset (13). However, applications to empirical systems can be appropriate and insightful as demonstrated with the likely hybrid origins and age of the wheat D subgenome from diploid wild relatives (14). There are other Bayesian implementations of the NMSC that use slightly different parametrization and priors to infer rooted ultrametric species networks from multilocus sequence alignments. The BEAST2 (15) add-on SpeciesNetwork (16) uses the birth-hybridization process, which models speciation, extinction, and hybridization rates under the NMSC, as a prior on the network. Another BEAST2 add-on SnappNet (17) estimates networks from biallelic markers. SnappNet extends Snapp (18) and is similar to `MCMC_BiMarkers` (11) in PhyloNet aside from the birth-hybridization process as the network prior and a more efficient likelihood computation (17). The proposed birth-death-hybridization process shows some improvements from the birth-hybridization process such that missing lineages do not have a large effect on the types of networks observed and that assuming more genetically similar individuals have a higher chance of hybridization can be a good idea for constraining search space (19). Bayesian methods can also be effective for estimating parameters such as divergence times on fixed networks, as implemented in BPP under the MSC with introgression (MSCi) model (20). Here, the species network topology is assumed and the posteriors of vertex ages, population sizes, and the inheritance probabilities are sampled using MCMC. While this does not search for the best network topology, it can be used to test hypotheses when the number of competing networks are small (21–23).

Network priors for Bayesian methods. Typically, a Poisson distribution for $p(\psi)$ and uniform distribution of [0,1] (or Beta distribution) for $p(\gamma)$ are considered appropriate as a network prior. The effect of the Poisson prior $p(\psi)$ mean value on the final result diminishes with the number of loci, but runtime increases with the mean value. `MCMC_SEQ` and `MCMC_BiMarkers`

use the alignment of multiple sequence or biallelic markers (suitable for those working with reference-based genotype data), respectively, and accommodate gene tree or genealogical uncertainty. Both methods require the same priors as in `MCMC_GT` but additionally specify a prior on the diameters of reticulation vertices, which is the number of branches between the parental lineages and their most recent common ancestor and should help from proposing too many reticulation edges between distant lineages where the chances of gene tree variation not caused by ILS or introgression is higher.

Starting trees. Many network analyses require a starting topology, which can be a randomly generated, one of the input gene trees, or an estimated species tree. The better fit to the data the starting topology has, the more helpful it becomes in the heuristic search of the optimal network. Methods in PhyloNet use a Minimizing Deep Coalescences (MDC) tree (24) by default. However, it is recommended to estimate the starting topology from the data using conventional methods, such as ASTRAL (25), BUCKy (26),*BEAST (27), or SVDQuartets (28), to start the search closer to the global optimum. When confidence in the starting topology is low, it is recommended to initiate multiple runs on multiple starting topologies to expand the heuristic search.

Allopolyploid networks from MUL-trees. Interestingly, the minimizing deep coalescence (MDC) criterion implemented in PhyloNet (29, 30) to estimate a maximum parsimony network, has been especially useful in inferring a network with allopolyploids. Such preference of using the MDC criterion over other optimality criteria stems from the computational efficiency, regardless of its undesirable accuracy as demonstrated in (31). (32) presents a straightforward and efficient pipeline that couples MDC criterion with a permutation scheme to reconstructs a multi-labeled tree (MUL-tree) from the set of estimated gene trees. A MUL-tree is a strictly bifurcating tree, but the same tip label could appear multiple times. While simply visualizing a MUL-tree can give some intuition regarding the hybrid origins of a polyploid species, post-processing of the resulting MUL-tree to a species network is possible using PADRE (33), Dendroscope (34). The workflow of obtaining networks from parsimony-based MUL-trees was extended in the Python package AllCoPol (35), which accounts for gene tree uncertainty and improved the optimization strategy over the original permutation approach. Most recently, Polyfest (36) performs both the MUL-tree construction and network estimation. Notably, Polyfest is robust to moderate amounts of ILS, whereas PADRE is not, and was able to recover the anticipated network for allohexaploid bread wheat despite Polyfest being a fast parsimony method compared to the full likelihood approach of the original study (37).

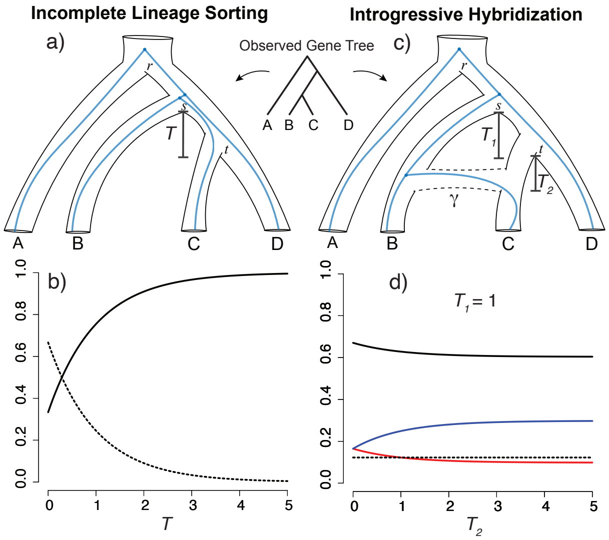

Fig. S1. There can be multiple explanations to causes of discordance between the observed gene tree and the underlying species tree. a) Incomplete lineage sorting (ILS) is pervasive and can cause variation due to sampling error or short intervals between speciation events. The multispecies coalescent model accommodates ILS, which derives the expected distribution of gene tree topologies based on the interval between speciation events T, measured in coalescent units. b) For a quartet, the probability (y-axis) that a gene tree matches the species tree (solid line) is $1 - \frac{2}{3}e^{-T}$ and the two discordant topologies occur in equal frequency (dotted line shows probability of both discordant topologies together) at $\frac{1}{3}e^{-T}$ (38). c) Phylogenetic network methods usually account for both ILS and hybridization, such that expected gene tree variation now depends on the time between speciation nodes T_1, the time between speciation and hybridization T_2, and the rate of episodic gene flow or the inheritance probability γ. Here, γ is the probability that sequences in lineage C trace back to a common ancestor with lineage B, while $1 - \gamma$ is the probability that sequences in lineage C trace back to a common ancestor with lineage D. d) The probabilities for each topology are given in (39), but and important observation can be made for even a single example where $T_1 = 1$ and $\gamma = 0.2$. The two discordant gene tree topologies no longer occur at equal frequencies. The gene tree placing the donor and receiving lineage of the hybridization event as sisters (blue) now occurs much more frequently than the other (red), which approaches the theoretical expectation under ILS alone (dotted line). In all panels, T is a coalescent unit measured as $\frac{t}{2N}$, where t is the number of generations and $2N$ is the diploid effective population size. Note that the effective population size is an expectation from genetic variation and not equivalent to the census population size.

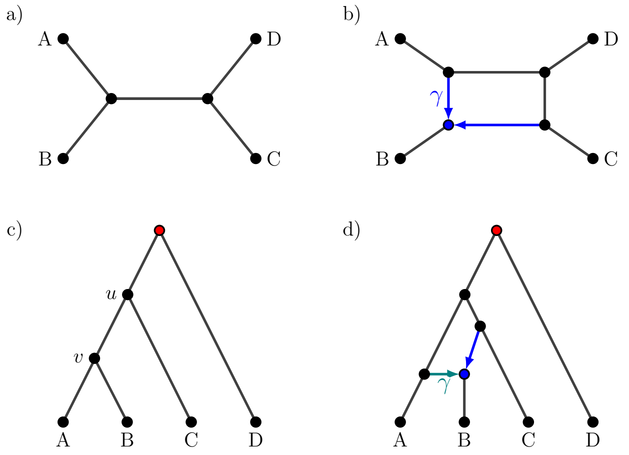

Fig. S2. Examples of unrooted and rooted trees and networks. a) An unrooted tree where the terminal nodes (or leaves) labeled with A, B, C, or D represent the sampled species. b) A semi-directed network with two reticulation edges (shown in blue) and a reticulation node (the ancestor of B). The inheritance probability, γ, is labeled for one of the reticulation edges, which makes the probability from the other edge $1 - \gamma$. c) A rooted tree with the root node shown in red. The directionality flows from the root towards the leaves, thus the node labeled as u in is interpreted as the ancestor of v. d) A rooted network, which depicts the direction of gene flow from A into B (shown in green) with inheritance probability γ. Notably, the branch length of the green edge is zero because A is an extant parental species of B

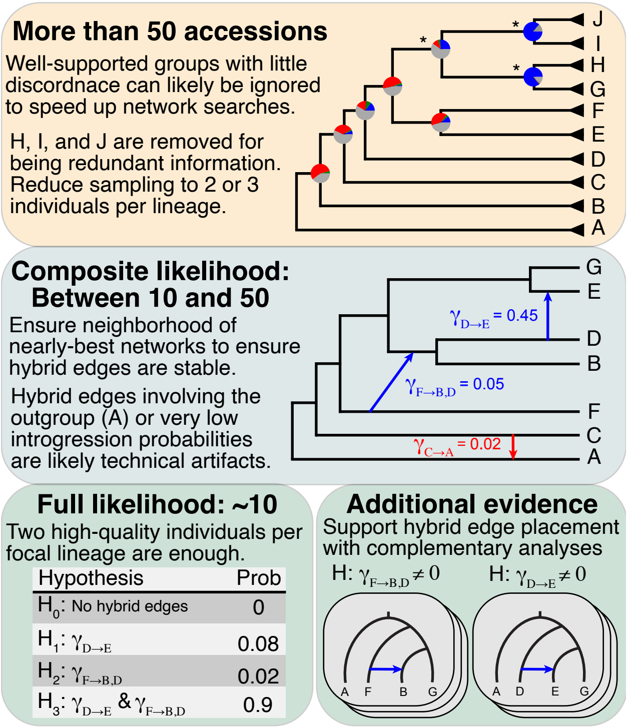

Fig. S3. Strategic sampling and design of network based investigations is possible within the context of much larger phylogenomic studies. Some parts of a larger phylogeny will have strong node support (marked by *) with little observable discordance between gene trees and species trees. Discordance analyses can be helpful for differentiating between strong conflict and a lack of information. Composite likelihood methods are then well-suited to network searches when the number of tips, meaning all individuals and not only the number of species, is not too high. Diligence is needed to avoid spurious reticulation edges as the models attempt to explain all gene tree variation through ILS and introgression. Other sources of gene tree variation such as technical errors or gene duplication and loss can pile up towards the root especially. Candidate networks or individual reticulation edges can then be validated using full likelihood methods or hybrid detection methods depending on the data and complexity of the hypotheses. Hybrid detection methods will not always be applicable to candidate reticulation edges depending on sampling, but can act as an extra check that candidate reticulation edges are plausible and not a false positive due to non-indentifiability of a more complex network.

Table S1. Common computational methods for phylogenetic network inference from genomic data.

Platform	Function/Algorithm/Model	Process	Optimality criterion	Input
NETRAX† (40)		Hybridization	ML	Partitioned MSA
PHYLONET	`InferNetwork_MP` (29)	Hybridization	MP	Gene trees
PHYLONET	`InferNetwork_ML` (41)	Hybridization	ML	Gene trees
PHYLONET	`MCMC_GT` (9)	Hybridization	Bayesian	Gene trees
PHYLONET	`MCMC_SEQ` (10)	Hybridization	Bayesian	MSA
PHYLONET	`MCMC_BiMarkers` (11)	Hybridization	Bayesian	Biallelic markers
PHYLONET	`InferNetwork_MPL` (42)	Hybridization	CL	Gene trees
PHYLONET	`MLE_BiMarkers` (43)	Hybridization	CL	Biallelic markers
PHYLONETWORKS	`SNaQ` (39)	Hybridization	CL	Gene trees‡
PHYNEST	`phyne!` (44)	Hybridization	CL	MSA
BEAST2	`SpeciesNetwork` (16)	Hybridization	Bayesian	MSA
BEAST2	`SnappNet` (17)	Hybridization	Bayesian	Biallelic markers
BPP	`MSCi` (20)	Introgression	Bayesian	MSA
NANUQ (45)		Hybridization	Combinatorial	Gene trees
RF-NET2† (46)		Reassortment and hybridization	Robinson-Fould distance	Gene trees
PADRE†(33)		Polyploidization	MP	MUL-trees
POLYPHEST(36)		Polyploidization		MUL-trees
*BEAST	`AlloppNET` (47–49)	Polyploidization	Bayesian	MSA
SOFTWARE PIPELINE	(32)	Polyploidization	MDC	Gene trees
PHYLONET	`InferNetwork_MP_Allopp` (50)	Polyploidization	MDC	Gene trees

† Assumes the incomplete lineage sorting is absent; ‡ other data types (e.g., single nucleotide polymorphism dataset) can be used to obtain the table of quartet concordance factors; ML: Maximum likelihood; MSA: Multiple sequence alignment; CL: Composite likelihood; MDC: Minimizing deep coalescence; MUL: Multi-labeled

References

1. LS Kubatko, J Chifman, An invariants-based method for efficient identification of hybrid species from large-scale genomic data. *BMC Evol. Biol.* **19**, 112 (2019).
2. N Patterson, et al., Ancient admixture in human history. *Genetics* **192**, 1065–1093 (2012).
3. MW Hahn, MS Hibbins, A three-sample test for introgression. *Mol Biol Evol* **36**, 2878–2882 (2019).
4. JAP Hamlin, MS Hibbins, LC Moyle, Assessing biological factors affecting postspeciation introgression. *Evol Lett* **4**, 137–154 (2020).
5. ES Allman, JD Mitchell, JA Rhodes, Gene tree discord, simplex plots, and statistical tests under the coalescent. *Syst. Biol.* **71**, 929–942 (2021).
6. JA Rhodes, H Baños, JD Mitchell, ES Allman, Mscquartets 1.0: quartet methods for species trees and networks under the multispecies coalescent model in R. *Bioinformatics* **37**, 1766–1768 (2021).
7. DA Baum, Concordance trees, concordance factors, and the exploration of reticulate genealogy. *TAXON* **56**, 417–426 (2007).
8. Z Wu, C Solís-Lemus, Ultrafast learning of four-node hybridization cycles in phylogenetic networks using algebraic invariants. *Bioinform Adv* **4**, vbae014 (2024).
9. D Wen, Y Yu, L Nakhleh, Bayesian inference of reticulate phylogenies under the multispecies network coalescent. *PLOS Genet.* **12**, e1006006 (2016).
10. D Wen, L Nakhleh, Coestimating reticulate phylogenies and gene trees from multilocus sequence data. *Syst. Biol.* **67**, 439–457 (2018).
11. J Zhu, D Wen, Y Yu, HM Meudt, L Nakhleh, Bayesian inference of phylogenetic networks from bi-allelic genetic markers. *PLOS Comput. Biol.* **14**, e1005932 (2018).
12. PO Lewis, MT Holder, KE Holsinger, Polytomies and Bayesian phylogenetic inference. *Syst. Biol.* **54**, 241–253 (2005).
13. MJ Sanderson, et al., Origin and diversification of the Saguaro cactus (*Carnegiea gigantea*): A within-species phylogenomic analysis. *Syst. Biol.* **71**, 1178–1194 (2022).
14. S Huynh, T Marcussen, F Felber, C Parisod, Hybridization preceded radiation in diploid wheats. *Mol. Phylogenetics Evol.* **139**, 106554 (2019).
15. R Bouckaert, et al., BEAST 2: A software platform for bayesian evolutionary analysis. *PLoS Comput. Biol.* **10**, e1003537 (2014).
16. C Zhang, HA Ogilvie, AJ Drummond, T Stadler, Bayesian inference of species networks from multilocus sequence data. *Mol. Biol. Evol.* **35**, 504–517 (2018).
17. CE Rabier, et al., On the inference of complex phylogenetic networks by Markov Chain Monte-Carlo. *PLOS Comput. Biol.* **17**, e1008380 (2021).
18. D Bryant, R Bouckaert, J Felsenstein, NA Rosenberg, A RoyChoudhury, Inferring species trees directly from biallelic genetic markers: Bypassing gene trees in a full coalescent analysis. *Mol. Biol. Evol.* **29**, 1917–1932 (2012).
19. JA Justison, TA Heath, Exploring the distribution of phylogenetic networks generated under a birth-death-hybridization process. *Bull. Soc. Syst. Biol.* **2** (2024).
20. T Flouri, X Jiao, B Rannala, Z Yang, A Bayesian implementation of the multispecies coalescent model with introgression for phylogenomic analysis. *Mol. Biol. Evol.* **37**, 1211–1223 (2020).
21. GP Tiley, et al., Estimation of species divergence times in presence of cross-species gene flow. *Syst. Biol.* **72**, 820–836 (2023).
22. GP Tiley, et al., Benefits and limits of phasing alleles for network inference of allopolyploid complexes. *Syst. Biol.* (2024) syae024.
23. B Fauskee, A Crowl, B Piatkowski, A Yoder, G Tiley, Ancient introgression in mouse lemurs (microcebus: Cheirogaleidae) explains 20 years of phylogenetic uncertainty. *Bull. Soc. Syst. Biol.* **3** (2024).
24. WP Maddison, Gene trees in species trees. *Syst. Biol.* **46**, 523–536 (1997).
25. C Zhang, M Rabiee, E Sayyari, S Mirarab, Astral-iii: polynomial time species tree reconstruction from partially resolved gene trees. *BMC Bioinforma.* **19**, 153 (2018).
26. BR Larget, SK Kotha, CN Dewey, C Ané, BUCKy: Gene tree/species tree reconciliation with Bayesian concordance analysis. *Bioinformatics* **26**, 2910–2911 (2010).
27. J Heled, AJ Drummond, Bayesian inference of species trees from multilocus data. *Mol. Biol. Evol.* **27**, 570–580 (2010).
28. J Chifman, L Kubatko, Quartet inference from SNP data under the coalescent model. *Bioinformatics* **30**, 3317–3324 (2014).
29. Y Yu, RM Barnett, L Nakhleh, Parsimonious inference of hybridization in the presence of incomplete lineage sorting. *Syst. Biol.* **62**, 738–751 (2013).
30. D Wen, Y Yu, J Zhu, L Nakhleh, Inferring phylogenetic networks using Phylonet. *Syst. Biol.* **67**, 735–740 (2018).
31. HA Hejase, KJ Liu, A scalability study of phylogenetic network inference methods using empirical datasets and simulations involving a single reticulation. *BMC Bioinforma.* **17**, 422 (2016).
32. C Oberprieler, F Wagner, S Tomasello, K Konowalik, A permutation approach for inferring species networks from gene trees in polyploid complexes by minimising deep coalescences. *Methods Ecol. Evol.* **8**, 835–849 (2017).
33. M Lott, A Spillner, KT Huber, V Moulton, PADRE: A package for analyzing and displaying reticulate evolution. *Bioinformatics* **25**, 1199–1200 (2009).

34. DH Huson, C Scornavacca, Dendroscope 3: An interactive tool for rooted phylogenetic trees and networks. *Syst. Biol.* **61**, 1061–1067 (2012).
35. U Lautenschlager, F Wagner, C Oberprieler, AllCoPol: Inferring allele co-ancestry in polyploids. *BMC Bioinforma.* **21**, 441 (2020).
36. Z Yan, Z Cao, L Nakhleh, Polyphest: fast polyploid phylogeny estimation. *Bioinformatics* **40**, ii20–ii28 (2024).
37. T Marcussen, et al., Ancient hybridizations among the ancestral genomes of bread wheat. *science* **345**, 1250092 (2014).
38. RR Hudson, Testing the constant-rate neutral allele model with protein sequence data. *Evolution* pp. 203–217 (1983).
39. C Solís-Lemus, C Ané, Inferring phylogenetic networks with maximum pseudolikelihood under incomplete lineage sorting. *PLOS Genet.* **12**, e1005896 (2016).
40. S Lutteropp, C Scornavacca, AM Kozlov, B Morel, A Stamatakis, NetRAX: Accurate and fast maximum likelihood phylogenetic network inference. *Bioinformatics* **38**, 3725–3733 (2022).
41. Y Yu, J Dong, KJ Liu, L Nakhleh, Maximum likelihood inference of reticulate evolutionary histories. *Proc. Natl. Acad. Sci.* **111**, 16448–16453 (2014).
42. Y Yu, L Nakhleh, A maximum pseudo-likelihood approach for phylogenetic networks. *BMC Genomics* **16**, S10 (2015).
43. J Zhu, L Nakhleh, Inference of species phylogenies from bi-allelic markers using pseudo-likelihood. *Bioinformatics* **34**, i376–i385 (2018).
44. S Kong, DL Swofford, LS Kubatko, Inference of phylogenetic networks from sequence data using composite likelihood. *bioRxiv* (2022).
45. ES Allman, H Baños, JD Mitchell, JA Rhodes, TINNiK: Inference of the tree of blobs of a species network under the coalescent. *bioRxiv* (2024).
46. A Markin, S Wagle, TK Anderson, O Eulenstein, RF-Net 2: Fast inference of virus reassortment and hybridization networks. *Bioinformatics* **38**, 2144–2152 (2022).
47. G Jones, S Sagitov, B Oxelman, Statistical inference of allopolyploid species networks in the presence of incomplete lineage sorting. *Syst. Biol.* **62**, 467–478 (2013).
48. G Jones, Bayesian phylogenetic analysis for diploid and allotetraploid species networks. *bioRxiv* (2017).
49. B Oxelman, et al., Phylogenetics of allopolyploids. *Annu. Rev. Ecol. Evol. Syst.* **48**, 543–557 (2017).
50. Z Yan, Z Cao, Y Liu, HA Ogilvie, L Nakhleh, Maximum parsimony inference of phylogenetic networks in the presence of polyploid complexes. *Syst. Biol.* **71**, 706–720 (2022).
